# *cis*-Dichloridobis(di-2-pyridyl­amine-κ^2^ *N*,*N*′)manganese(II)

**DOI:** 10.1107/S1600536811052834

**Published:** 2011-12-14

**Authors:** Kwang Ha

**Affiliations:** a School of Applied Chemical Engineering, The Research Institute of Catalysis, Chonnam National University, Gwangju 500-757, Republic of Korea

## Abstract

In the title complex, [MnCl_2_(C_10_H_9_N_3_)_2_], the Mn^II^ ion is six-coordinated in a considerably distorted *cis*-N_4_Cl_2_ octa­hedral environment defined by four N atoms of two chelating di-2-pyridyl­amine (dpa) ligands and two Cl^−^ anions. In the crystal, the dpa ligands are not planar, the dihedral angles between the two pyridine rings being 29.3 (2) and 30.9 (2)°. The complex mol­ecules are stacked in columns along the *c* axis and are connected by inter­molecular N—H⋯Cl hydrogen bonds, forming a three-dimensional network. Weak inter- and intra­molecular π–π inter­actions are present between the pyridine rings, the shortest centroid—centroid distance being 4.406 (3) Å.

## Related literature

For the crystal structures of related Mn^II^ complexes with dpa, see: Bose *et al.* (2005 ▶); Ha (2011*a* ▶,*b* ▶).
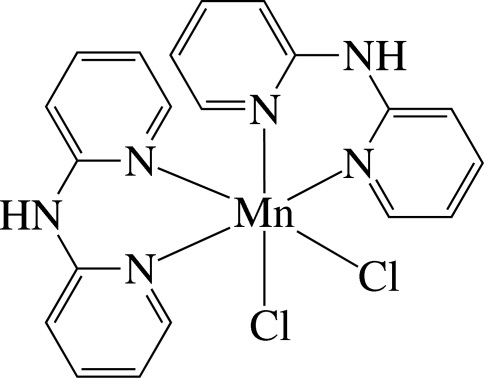


## Experimental

### 

#### Crystal data

[MnCl_2_(C_10_H_9_N_3_)_2_]
*M* *_r_* = 468.24
Orthorhombic, 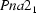

*a* = 16.236 (3) Å
*b* = 12.542 (2) Å
*c* = 9.9233 (17) Å
*V* = 2020.7 (6) Å^3^
*Z* = 4
Mo *K*α radiation
μ = 0.94 mm^−1^
*T* = 200 K
0.31 × 0.28 × 0.19 mm

#### Data collection

Bruker SMART 1000 CCD diffractometer
Absorption correction: multi-scan (*SADABS*; Bruker, 2000 ▶) *T*_min_ = 0.849, *T*_max_ = 1.000
14151 measured reflections
4293 independent reflections
2982 reflections with *I* > 2σ(*I*)
*R*_int_ = 0.072

#### Refinement

*R*[*F* ^2^ > 2σ(*F* ^2^)] = 0.042
*wR*(*F* ^2^) = 0.092
*S* = 1.01
4293 reflections
262 parameters
1 restraint
H-atom parameters constrained
Δρ_max_ = 0.51 e Å^−3^
Δρ_min_ = −0.57 e Å^−3^
Absolute structure: Flack (1983 ▶), 1616 Friedel pairs
Flack parameter: 0.04 (2)

### 

Data collection: *SMART* (Bruker, 2000 ▶); cell refinement: *SAINT* (Bruker, 2000 ▶); data reduction: *SAINT*; program(s) used to solve structure: *SHELXS97* (Sheldrick, 2008 ▶); program(s) used to refine structure: *SHELXL97* (Sheldrick, 2008 ▶); molecular graphics: *ORTEP-3* (Farrugia, 1997 ▶) and *PLATON* (Spek, 2009 ▶); software used to prepare material for publication: *SHELXL97*.

## Supplementary Material

Crystal structure: contains datablock(s) global, I. DOI: 10.1107/S1600536811052834/wm2573sup1.cif

Structure factors: contains datablock(s) I. DOI: 10.1107/S1600536811052834/wm2573Isup2.hkl

Additional supplementary materials:  crystallographic information; 3D view; checkCIF report

## Figures and Tables

**Table:** 

Mn1—N3	2.276 (3)
Mn1—N1	2.278 (3)
Mn1—N4	2.280 (3)
Mn1—N6	2.353 (3)
Mn1—Cl2	2.4637 (12)
Mn1—Cl1	2.5122 (10)

**Table:** 

N3—Mn1—N1	77.32 (13)
N4—Mn1—N6	77.12 (12)

**Table 2:** Hydrogen-bond geometry (Å, °)

*D*—H⋯*A*	*D*—H	H⋯*A*	*D*⋯*A*	*D*—H⋯*A*
N2—H2*N*⋯Cl2^i^	0.92	2.30	3.211 (3)	171
N5—H5*N*⋯Cl1^ii^	0.92	2.45	3.355 (4)	170

Symmetry codes: (i) 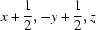; (ii) 
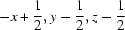
.

**Fig. 1.**
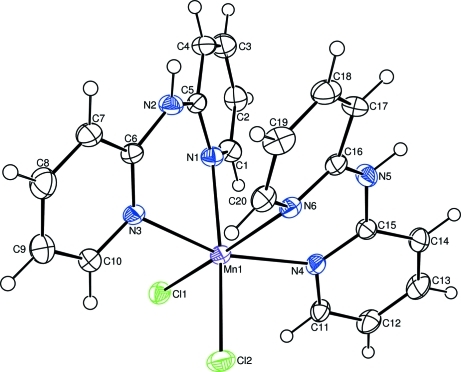
The molecular structure of the title complex, with displacement ellipsoids drawn at the 40% probability level for non-H atoms.

**Fig. 2.**
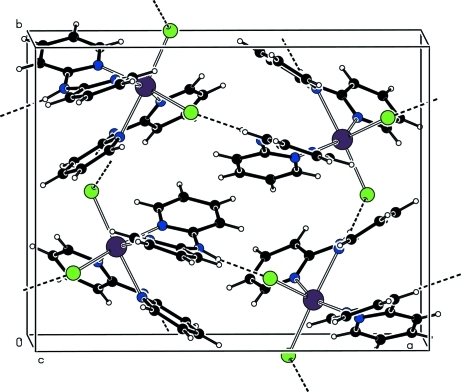
View of the unit-cell contents of the title complex. Hydrogen-bonding interactions are drawn with dashed lines.

## supplementary crystallographic information

### Comment

Neutral and cationic Mn^II^ complexes of the di-2-pyridylamine (dpa; C_10_H_9_N_3_) ligand, such as [Mn*X*_2_(dpa)_2_]^.^H_2_O, [Mn*X*(dpa)_2_(H_2_O)]ClO_4_ (*X* = N_3_^-^, NCO^-^) (Bose *et al.*, 2005) and [Mn*X*(dpa)_2_(H_2_O)]*X* (*X* = I, Br) (Ha, 2011*a*,*b*), have been investigated previously.

In the title complex, [MnCl_2_(dpa)_2_], the Mn^II^ ion is six-coordinated in a considerably distorted *cis*-N_4_Cl_2_ octahedral environment defined by four N atoms of two chelating dpa ligands and two Cl^-^ anions (Fig. 1). The main contributions to the distortion are the tight N—Mn—N chelating angles (Table 1), which results in non-linear *trans* axes [N3—Mn1—N4 = 161.55 (11)°, N6—Mn1—Cl1 = 173.54 (11)° and N1—Mn1—Cl2 = 170.18 (10)°]. Because the Mn—N bond lengths are nearly equivalent (Table 1), the different *trans* effects of the Cl and N atoms cannot be observed reliably. In the crystal structure, the dpa ligands are not planar, the dihedral angles between the two pyridine rings being 29.3 (2)° and 30.9 (2)°. The complex molecules are stacked in columns along the *c* axis and connected by intermolecular N—H···Cl hydrogen bonds, forming a three-dimensional network (Fig. 2, Table 2). In the columns, numerous weak inter- and intramolecular π—π interactions are present between the pyridine rings, the shortest centroid-centroid distance being 4.406 (3) Å.

### Experimental

To a solution of MnCl_2_.4H_2_O (0.1988 g, 1.005 mmol) in EtOH (20 ml) was added di-2-pyridylamine (0.3465 g, 2.024 mmol) and stirred for 3 h at room temperature. The formed precipitate was separated by filtration and washed with EtOH and acetone, and dried at 323 K, to give a white powder (0.2982 g). Crystals suitable for X-ray analysis were obtained by slow evaporation from a CH_3_CN solution.

### Refinement

Carbon-bound H atoms were positioned geometrically and allowed to ride on their respective parent atoms [C—H = 0.95 Å and *U*_iso_(H) = 1.2*U*_eq_(C)]. Nitrogen-bound H atoms were located from Fourier difference maps then allowed to ride on their parent atoms in the final cycles of refinement with N—H = 0.92 Å and *U*_iso_(H) = 1.5 *U*_eq_(N). The highest peak (0.51 e Å^-3^) and the deepest hole (-0.56 e Å^-3^) in the difference Fourier map are located 1.40 Å and 1.08 Å from the atoms H9 and N4, respectively.

### Crystal data

**Table:**

[MnCl_2_(C_10_H_9_N_3_)_2_]	*F*(000) = 956
*M**_r_* = 468.24	*D*_x_ = 1.539 Mg m^−^^3^
Orthorhombic, *P**n**a*2_1_	Mo *K*α radiation, λ = 0.71073 Å
Hall symbol: P 2c -2n	Cell parameters from 4041 reflections
*a* = 16.236 (3) Å	θ = 2.5–27.8°
*b* = 12.542 (2) Å	µ = 0.94 mm^−^^1^
*c* = 9.9233 (17) Å	*T* = 200 K
*V* = 2020.7 (6) Å^3^	Block, colorless
*Z* = 4	0.31 × 0.28 × 0.19 mm

### Data collection

**Table:**

Bruker SMART 1000 CCD diffractometer	4293 independent reflections
Radiation source: fine-focus sealed tube	2982 reflections with *I* > 2σ(*I*)
graphite	*R*_int_ = 0.072
φ and ω scans	θ_max_ = 28.4°, θ_min_ = 2.1°
Absorption correction: multi-scan (*SADABS*; Bruker, 2000)	*h* = −21→21
*T*_min_ = 0.849, *T*_max_ = 1.000	*k* = −16→15
14151 measured reflections	*l* = −13→9

### Refinement

**Table:**

Refinement on *F*^2^	Secondary atom site location: difference Fourier map
Least-squares matrix: full	Hydrogen site location: inferred from neighbouring sites
*R*[*F*^2^ > 2σ(*F*^2^)] = 0.042	H-atom parameters constrained
*wR*(*F*^2^) = 0.092	*w* = 1/[σ^2^(*F*_o_^2^) + (0.0271*P*)^2^] where *P* = (*F*_o_^2^ + 2*F*_c_^2^)/3
*S* = 1.01	(Δ/σ)_max_ < 0.001
4293 reflections	Δρ_max_ = 0.51 e Å^−^^3^
262 parameters	Δρ_min_ = −0.57 e Å^−^^3^
1 restraint	Absolute structure: Flack (1983), 1616 Friedel pairs
Primary atom site location: structure-invariant direct methods	Flack parameter: 0.04 (2)

### Special details

**Table:**

Geometry. All e.s.d.'s (except the e.s.d. in the dihedral angle between two l.s. planes) are estimated using the full covariance matrix. The cell e.s.d.'s are taken into account individually in the estimation of e.s.d.'s in distances, angles and torsion angles; correlations between e.s.d.'s in cell parameters are only used when they are defined by crystal symmetry. An approximate (isotropic) treatment of cell e.s.d.'s is used for estimating e.s.d.'s involving l.s. planes.
Refinement. Refinement of *F*^2^ against ALL reflections. The weighted *R*-factor *wR* and goodness of fit *S* are based on *F*^2^, conventional *R*-factors *R* are based on *F*, with *F* set to zero for negative *F*^2^. The threshold expression of *F*^2^ > σ(*F*^2^) is used only for calculating *R*-factors(gt) *etc*. and is not relevant to the choice of reflections for refinement. *R*-factors based on *F*^2^ are statistically about twice as large as those based on *F*, and *R*- factors based on ALL data will be even larger.

### Fractional atomic coordinates and isotropic or equivalent isotropic displacement parameters (Å^2^)

**Table:**

	*x*	*y*	*z*	*U*_iso_*/*U*_eq_	
Mn1	0.20798 (3)	0.33815 (4)	0.89348 (7)	0.02448 (14)	
Cl1	0.14448 (5)	0.52067 (7)	0.88475 (14)	0.0317 (2)	
Cl2	0.09525 (6)	0.25950 (9)	1.02667 (13)	0.0376 (3)	
N1	0.3262 (2)	0.4028 (3)	0.8006 (4)	0.0270 (8)	
N2	0.4152 (2)	0.3360 (3)	0.9693 (4)	0.0293 (8)	
H2N	0.4643	0.3016	0.9842	0.044*	
N3	0.28805 (19)	0.3701 (2)	1.0774 (3)	0.0251 (8)	
N4	0.16475 (18)	0.2772 (2)	0.6889 (4)	0.0285 (8)	
N5	0.2814 (2)	0.1689 (3)	0.6387 (4)	0.0301 (8)	
H5N	0.3074	0.1343	0.5690	0.045*	
N6	0.27298 (17)	0.1710 (2)	0.8770 (4)	0.0246 (7)	
C1	0.3191 (3)	0.4552 (3)	0.6826 (5)	0.0330 (10)	
H1	0.2652	0.4722	0.6518	0.040*	
C2	0.3842 (3)	0.4853 (3)	0.6045 (5)	0.0363 (11)	
H2	0.3758	0.5225	0.5222	0.044*	
C3	0.4629 (3)	0.4602 (3)	0.6481 (5)	0.0421 (12)	
H3	0.5095	0.4787	0.5950	0.051*	
C4	0.4730 (2)	0.4089 (3)	0.7678 (5)	0.0346 (11)	
H4	0.5265	0.3909	0.7991	0.042*	
C5	0.4036 (2)	0.3830 (3)	0.8440 (4)	0.0266 (10)	
C6	0.3676 (2)	0.3427 (3)	1.0850 (4)	0.0257 (9)	
C7	0.4062 (3)	0.3193 (3)	1.2082 (4)	0.0356 (11)	
H7	0.4618	0.2956	1.2100	0.043*	
C8	0.3632 (3)	0.3312 (3)	1.3248 (5)	0.0410 (11)	
H8	0.3879	0.3145	1.4090	0.049*	
C9	0.2820 (3)	0.3684 (4)	1.3191 (5)	0.0393 (12)	
H9	0.2519	0.3827	1.3992	0.047*	
C10	0.2472 (2)	0.3836 (3)	1.1952 (5)	0.0318 (10)	
H10	0.1911	0.4048	1.1915	0.038*	
C11	0.0903 (2)	0.3143 (3)	0.6470 (4)	0.0294 (10)	
H11	0.0612	0.3608	0.7056	0.035*	
C12	0.0546 (3)	0.2892 (3)	0.5268 (5)	0.0386 (11)	
H12	0.0029	0.3186	0.5020	0.046*	
C13	0.0957 (3)	0.2196 (4)	0.4416 (5)	0.0446 (13)	
H13	0.0726	0.2011	0.3567	0.054*	
C14	0.1699 (3)	0.1775 (3)	0.4811 (5)	0.0361 (11)	
H14	0.1984	0.1287	0.4246	0.043*	
C15	0.2032 (2)	0.2078 (3)	0.6067 (4)	0.0261 (9)	
C16	0.3129 (3)	0.1420 (3)	0.7646 (5)	0.0256 (10)	
C17	0.3864 (2)	0.0827 (3)	0.7674 (5)	0.0302 (11)	
H17	0.4141	0.0650	0.6861	0.036*	
C18	0.4171 (2)	0.0510 (3)	0.8890 (6)	0.0369 (10)	
H18	0.4680	0.0139	0.8936	0.044*	
C19	0.3733 (3)	0.0735 (4)	1.0062 (5)	0.0366 (12)	
H19	0.3918	0.0487	1.0914	0.044*	
C20	0.3017 (3)	0.1336 (3)	0.9945 (5)	0.0298 (11)	
H20	0.2715	0.1489	1.0742	0.036*	

### Atomic displacement parameters (Å^2^)

**Table:**

	*U*^11^	*U*^22^	*U*^33^	*U*^12^	*U*^13^	*U*^23^
Mn1	0.0197 (3)	0.0278 (3)	0.0260 (3)	0.0000 (2)	−0.0022 (3)	−0.0011 (4)
Cl1	0.0290 (5)	0.0306 (5)	0.0355 (6)	0.0023 (4)	−0.0053 (6)	−0.0022 (6)
Cl2	0.0253 (5)	0.0455 (6)	0.0421 (6)	−0.0082 (5)	0.0032 (5)	0.0019 (6)
N1	0.0235 (18)	0.0270 (18)	0.031 (2)	0.0015 (14)	0.0000 (15)	0.0022 (16)
N2	0.0211 (18)	0.038 (2)	0.029 (2)	0.0048 (15)	−0.0009 (16)	−0.0002 (17)
N3	0.0195 (18)	0.0309 (18)	0.025 (2)	0.0009 (14)	−0.0034 (15)	−0.0008 (16)
N4	0.0236 (18)	0.0278 (18)	0.034 (2)	0.0006 (14)	−0.0041 (16)	−0.0060 (16)
N5	0.0285 (19)	0.037 (2)	0.024 (2)	0.0072 (15)	−0.0035 (16)	−0.0052 (16)
N6	0.0213 (15)	0.0248 (15)	0.028 (2)	−0.0013 (12)	−0.0028 (17)	0.0002 (18)
C1	0.035 (2)	0.033 (2)	0.032 (3)	0.0018 (18)	0.003 (2)	0.003 (2)
C2	0.048 (3)	0.033 (2)	0.028 (3)	−0.005 (2)	0.006 (2)	0.005 (2)
C3	0.042 (3)	0.041 (3)	0.043 (3)	−0.012 (2)	0.014 (2)	0.001 (2)
C4	0.023 (2)	0.037 (2)	0.044 (3)	−0.0021 (18)	0.005 (2)	−0.004 (2)
C5	0.022 (2)	0.025 (2)	0.033 (3)	−0.0015 (17)	−0.0012 (18)	−0.0053 (18)
C6	0.020 (2)	0.028 (2)	0.028 (2)	0.0009 (16)	−0.0025 (18)	−0.0011 (19)
C7	0.026 (2)	0.048 (3)	0.033 (3)	0.0042 (19)	−0.005 (2)	−0.005 (2)
C8	0.044 (3)	0.048 (3)	0.031 (3)	0.002 (2)	−0.008 (2)	−0.003 (2)
C9	0.043 (3)	0.046 (3)	0.029 (3)	−0.001 (2)	−0.001 (2)	−0.005 (2)
C10	0.028 (2)	0.034 (2)	0.033 (3)	0.0005 (18)	0.001 (2)	−0.007 (2)
C11	0.028 (2)	0.029 (2)	0.031 (3)	0.0005 (17)	−0.0078 (19)	−0.004 (2)
C12	0.029 (2)	0.038 (2)	0.048 (3)	0.0030 (19)	−0.014 (2)	0.000 (2)
C13	0.036 (3)	0.057 (3)	0.041 (3)	−0.002 (2)	−0.020 (2)	−0.009 (2)
C14	0.035 (3)	0.044 (3)	0.029 (3)	0.000 (2)	−0.006 (2)	−0.011 (2)
C15	0.025 (2)	0.028 (2)	0.025 (2)	0.0007 (16)	−0.0013 (18)	0.0000 (19)
C16	0.023 (2)	0.029 (2)	0.025 (3)	−0.0015 (18)	−0.0042 (19)	0.000 (2)
C17	0.021 (2)	0.035 (3)	0.035 (3)	0.0047 (18)	0.003 (2)	0.000 (2)
C18	0.027 (2)	0.034 (2)	0.050 (3)	0.0023 (16)	−0.003 (3)	0.004 (3)
C19	0.034 (3)	0.037 (3)	0.039 (3)	−0.001 (2)	−0.005 (2)	0.013 (2)
C20	0.033 (3)	0.022 (2)	0.035 (3)	−0.0028 (19)	−0.003 (2)	0.004 (2)

### Geometric parameters (Å, °)

**Table:**

Mn1—N3	2.276 (3)	C4—C5	1.395 (5)
Mn1—N1	2.278 (3)	C4—H4	0.9500
Mn1—N4	2.280 (3)	C6—C7	1.405 (6)
Mn1—N6	2.353 (3)	C7—C8	1.360 (6)
Mn1—Cl2	2.4637 (12)	C7—H7	0.9500
Mn1—Cl1	2.5122 (10)	C8—C9	1.399 (6)
N1—C1	1.348 (5)	C8—H8	0.9500
N1—C5	1.351 (5)	C9—C10	1.367 (6)
N2—C6	1.386 (5)	C9—H9	0.9500
N2—C5	1.389 (5)	C10—H10	0.9500
N2—H2N	0.9200	C11—C12	1.363 (6)
N3—C6	1.339 (4)	C11—H11	0.9500
N3—C10	1.355 (5)	C12—C13	1.386 (6)
N4—C15	1.346 (5)	C12—H12	0.9500
N4—C11	1.360 (5)	C13—C14	1.373 (6)
N5—C16	1.392 (6)	C13—H13	0.9500
N5—C15	1.396 (5)	C14—C15	1.411 (6)
N5—H5N	0.9200	C14—H14	0.9500
N6—C16	1.340 (6)	C16—C17	1.405 (6)
N6—C20	1.341 (6)	C17—C18	1.365 (7)
C1—C2	1.365 (6)	C17—H17	0.9500
C1—H1	0.9500	C18—C19	1.392 (7)
C2—C3	1.385 (6)	C18—H18	0.9500
C2—H2	0.9500	C19—C20	1.389 (6)
C3—C4	1.361 (6)	C19—H19	0.9500
C3—H3	0.9500	C20—H20	0.9500
			
N3—Mn1—N1	77.32 (13)	N2—C5—C4	118.3 (4)
N3—Mn1—N4	161.55 (11)	N3—C6—N2	120.4 (4)
N1—Mn1—N4	91.06 (12)	N3—C6—C7	122.2 (4)
N3—Mn1—N6	87.50 (12)	N2—C6—C7	117.4 (3)
N1—Mn1—N6	84.90 (11)	C8—C7—C6	119.2 (4)
N4—Mn1—N6	77.12 (12)	C8—C7—H7	120.4
N3—Mn1—Cl2	93.71 (9)	C6—C7—H7	120.4
N1—Mn1—Cl2	170.18 (10)	C7—C8—C9	119.1 (4)
N4—Mn1—Cl2	96.59 (9)	C7—C8—H8	120.4
N6—Mn1—Cl2	90.79 (9)	C9—C8—H8	120.4
N3—Mn1—Cl1	95.84 (8)	C10—C9—C8	118.2 (4)
N1—Mn1—Cl1	90.43 (9)	C10—C9—H9	120.9
N4—Mn1—Cl1	98.55 (9)	C8—C9—H9	120.9
N6—Mn1—Cl1	173.54 (11)	N3—C10—C9	123.8 (4)
Cl2—Mn1—Cl1	94.50 (4)	N3—C10—H10	118.1
C1—N1—C5	116.5 (4)	C9—C10—H10	118.1
C1—N1—Mn1	116.9 (3)	N4—C11—C12	124.5 (4)
C5—N1—Mn1	126.1 (3)	N4—C11—H11	117.8
C6—N2—C5	129.8 (3)	C12—C11—H11	117.8
C6—N2—H2N	112.2	C11—C12—C13	118.4 (4)
C5—N2—H2N	117.4	C11—C12—H12	120.8
C6—N3—C10	117.1 (3)	C13—C12—H12	120.8
C6—N3—Mn1	123.5 (3)	C14—C13—C12	119.4 (4)
C10—N3—Mn1	115.7 (3)	C14—C13—H13	120.3
C15—N4—C11	116.7 (3)	C12—C13—H13	120.3
C15—N4—Mn1	127.8 (3)	C13—C14—C15	119.0 (4)
C11—N4—Mn1	115.5 (3)	C13—C14—H14	120.5
C16—N5—C15	128.6 (4)	C15—C14—H14	120.5
C16—N5—H5N	113.0	N4—C15—N5	120.6 (4)
C15—N5—H5N	114.3	N4—C15—C14	122.1 (4)
C16—N6—C20	117.4 (3)	N5—C15—C14	117.1 (4)
C16—N6—Mn1	121.1 (3)	N6—C16—N5	120.2 (4)
C20—N6—Mn1	114.0 (3)	N6—C16—C17	122.5 (4)
N1—C1—C2	124.2 (4)	N5—C16—C17	117.3 (4)
N1—C1—H1	117.9	C18—C17—C16	118.8 (4)
C2—C1—H1	117.9	C18—C17—H17	120.6
C1—C2—C3	118.3 (4)	C16—C17—H17	120.6
C1—C2—H2	120.8	C17—C18—C19	119.5 (3)
C3—C2—H2	120.8	C17—C18—H18	120.2
C4—C3—C2	119.4 (4)	C19—C18—H18	120.2
C4—C3—H3	120.3	C20—C19—C18	117.9 (5)
C2—C3—H3	120.3	C20—C19—H19	121.1
C3—C4—C5	119.1 (4)	C18—C19—H19	121.1
C3—C4—H4	120.5	N6—C20—C19	123.6 (5)
C5—C4—H4	120.5	N6—C20—H20	118.2
N1—C5—N2	119.3 (4)	C19—C20—H20	118.2
N1—C5—C4	122.4 (4)		
			
N3—Mn1—N1—C1	−150.8 (3)	Mn1—N1—C5—C4	167.7 (3)
N4—Mn1—N1—C1	43.7 (3)	C6—N2—C5—N1	−30.3 (6)
N6—Mn1—N1—C1	120.6 (3)	C6—N2—C5—C4	149.2 (4)
Cl1—Mn1—N1—C1	−54.9 (3)	C3—C4—C5—N1	3.1 (6)
N3—Mn1—N1—C5	37.5 (3)	C3—C4—C5—N2	−176.5 (4)
N4—Mn1—N1—C5	−128.0 (3)	C10—N3—C6—N2	−174.7 (4)
N6—Mn1—N1—C5	−51.1 (3)	Mn1—N3—C6—N2	28.0 (5)
Cl1—Mn1—N1—C5	133.4 (3)	C10—N3—C6—C7	5.6 (6)
N1—Mn1—N3—C6	−44.8 (3)	Mn1—N3—C6—C7	−151.8 (3)
N4—Mn1—N3—C6	7.3 (6)	C5—N2—C6—N3	21.9 (6)
N6—Mn1—N3—C6	40.6 (3)	C5—N2—C6—C7	−158.3 (4)
Cl2—Mn1—N3—C6	131.2 (3)	N3—C6—C7—C8	−4.2 (6)
Cl1—Mn1—N3—C6	−133.9 (3)	N2—C6—C7—C8	176.0 (4)
N1—Mn1—N3—C10	157.6 (3)	C6—C7—C8—C9	−1.3 (7)
N4—Mn1—N3—C10	−150.4 (3)	C7—C8—C9—C10	4.9 (7)
N6—Mn1—N3—C10	−117.1 (3)	C6—N3—C10—C9	−1.6 (6)
Cl2—Mn1—N3—C10	−26.5 (3)	Mn1—N3—C10—C9	157.5 (4)
Cl1—Mn1—N3—C10	68.4 (3)	C8—C9—C10—N3	−3.6 (7)
N3—Mn1—N4—C15	7.8 (6)	C15—N4—C11—C12	−2.7 (6)
N1—Mn1—N4—C15	58.1 (3)	Mn1—N4—C11—C12	178.5 (3)
N6—Mn1—N4—C15	−26.4 (3)	N4—C11—C12—C13	1.3 (7)
Cl2—Mn1—N4—C15	−115.7 (3)	C11—C12—C13—C14	0.7 (7)
Cl1—Mn1—N4—C15	148.7 (3)	C12—C13—C14—C15	−1.1 (7)
N3—Mn1—N4—C11	−173.6 (3)	C11—N4—C15—N5	177.4 (4)
N1—Mn1—N4—C11	−123.3 (3)	Mn1—N4—C15—N5	−4.1 (5)
N6—Mn1—N4—C11	152.2 (3)	C11—N4—C15—C14	2.2 (6)
Cl2—Mn1—N4—C11	62.9 (3)	Mn1—N4—C15—C14	−179.2 (3)
Cl1—Mn1—N4—C11	−32.7 (3)	C16—N5—C15—N4	37.4 (6)
N3—Mn1—N6—C16	−122.6 (3)	C16—N5—C15—C14	−147.1 (4)
N1—Mn1—N6—C16	−45.1 (3)	C13—C14—C15—N4	−0.4 (6)
N4—Mn1—N6—C16	47.1 (3)	C13—C14—C15—N5	−175.7 (4)
Cl2—Mn1—N6—C16	143.7 (3)	C20—N6—C16—N5	173.4 (4)
N3—Mn1—N6—C20	26.5 (3)	Mn1—N6—C16—N5	−38.6 (5)
N1—Mn1—N6—C20	103.9 (3)	C20—N6—C16—C17	−5.6 (5)
N4—Mn1—N6—C20	−163.8 (3)	Mn1—N6—C16—C17	142.5 (3)
Cl2—Mn1—N6—C20	−67.2 (3)	C15—N5—C16—N6	−12.8 (6)
C5—N1—C1—C2	2.3 (6)	C15—N5—C16—C17	166.2 (4)
Mn1—N1—C1—C2	−170.2 (3)	N6—C16—C17—C18	1.8 (6)
N1—C1—C2—C3	0.4 (6)	N5—C16—C17—C18	−177.2 (4)
C1—C2—C3—C4	−1.4 (6)	C16—C17—C18—C19	3.0 (6)
C2—C3—C4—C5	−0.2 (6)	C17—C18—C19—C20	−3.7 (6)
C1—N1—C5—N2	175.5 (4)	C16—N6—C20—C19	4.8 (5)
Mn1—N1—C5—N2	−12.7 (5)	Mn1—N6—C20—C19	−145.5 (3)
C1—N1—C5—C4	−4.0 (6)	C18—C19—C20—N6	−0.2 (6)

### Hydrogen-bond geometry (Å, °)

**Table:**

*D*—H···*A*	*D*—H	H···*A*	*D*···*A*	*D*—H···*A*
N2—H2N···Cl2^i^	0.92	2.30	3.211 (3)	171.
N5—H5N···Cl1^ii^	0.92	2.45	3.355 (4)	170.

Symmetry codes: (i) *x*+1/2, −*y*+1/2, *z*; (ii) −*x*+1/2, *y*−1/2, *z*−1/2.
